# Combining Visual Feedback and Noninvasive Brain Stimulation for Lower Limb Motor Rehabilitation in Stroke: A Systematic Review of the Current Evidence

**DOI:** 10.3390/jcm14145027

**Published:** 2025-07-16

**Authors:** Leonardo Di Cosmo, Santiago Nieto Cuervo, Francesca Pellicanò, Francesca Romana Centini, Jad El Choueiri, Chiara Learmonth, Filippo Emanuele Colella, Lorenzo De Rossi, Delia Cannizzaro, Alessio Baricich

**Affiliations:** 1 School of Medicine, Humanitas University, Pieve Emanuele, 20072 Milan, Italy; 2 Department of Neurosurgery, ASST Ovest Milano Legnano Hospital, Legnano, 20025 Milan, Italy; 3 Department of Biomedical Sciences, Humanitas University, Pieve Emanuele, 20072 Milan, Italy; 4 Rehabilitation Unit, IRCCS Humanitas Research Hospital, Rozzano, 20089 Milan, Italy

**Keywords:** stroke, lower limb rehabilitation, motor rehabilitation, noninvasive brain stimulation, visual feedback

## Abstract

**Background and Objectives:** Recent technological advances have introduced new interventions in the field of stroke rehabilitation. Among them, visual feedback (VF) and non-invasive brain stimulation (NIBS) have gained considerable attention, with growing evidence supporting their efficacy. However, their combined application in lower limb recovery remains to be established. This systematic review aims to evaluate the current evidence on the therapeutic effect of combining VF and NIBS for lower limb motor rehabilitation in stroke patients. **Methods:** Following PRISMA guidelines, PubMed, Embase, Scopus, and Cochrane databases were searched for randomized controlled trials and observational studies comparing VF and NIBS interventions with either their monotherapy, placebo, or standard treatment. The outcomes evaluated for lower limb function included balance, gait, and motor performance. **Results:** From 997 studies screened, 5 studies (3 RCTs and 2 cohort studies) were included. Despite heterogeneity in the immersion level, NIBS protocols, and outcome measures, evidence emerged supporting the efficacy of combined VF and NIBS across multiple outcomes. However, the degree to which these interventions outperform standard therapies remains uncertain, primarily due to a limited number of comparator studies and the quality of the existing data. **Conclusions:** This review provides preliminary insights into the potential of combining VF and NIBS in stroke patients affected by lower limb motor impairments. Future research should focus on standardizing protocols and addressing demographic variability to enhance the reliability and comparability of findings.

## 1. Introduction

Stroke is one of the leading causes of long-term disability worldwide, with motor dysfunction affecting approximately 85% of survivors [1,2,3]. Data from 800,000 U.S. stroke patients demonstrated that 25% experienced severe lower extremity paresis [4]. Lower limb motor impairment is a primary driver of disability and is closely linked to poorer quality of life and mental wellbeing within this population [5,6].

Various factors, including training intensity, task specificity, patient motivation, and the quality of feedback, influence the efficacy of motor rehabilitation in stroke patients [7]. Traditional rehabilitation methods focus on movement repetition and intensity, which do not exploit direct neuroplastic targeting, and in many cases, fulfill patient needs [8].

Recent therapeutic developments have shifted toward actively mediating synaptic plasticity through non-invasive brain stimulation (NIBS) [9,10]. NIBS includes techniques such as transcranial magnetic stimulation (TMS) and transcranial direct current stimulation (tDCS), which modulate cortical excitability through the application of magnetic and electrical currents, respectively [11,12]. These devices have been demonstrated not only to improve motor function but also cognitive domains, such as hemispatial neglect and post-stroke memory impairment [13,14].

Visual feedback (VF) is a central component in motor rehabilitation protocols, providing real-time sensory feedback to movement patterns. Contemporary VF systems cover a spectrum of technologies, including virtual reality (VR), mirror therapy, and brain–computer interfaces (BCIs). These can be broadly categorized into immersive systems, which integrate digital and real-world environments through interactive feedback and non-immersive systems, such as mirror therapy, which rely on more static visual cues [14,15].

While both NIBS and VF have independently demonstrated therapeutic benefits, their combined application has not been established. A recent meta-analysis on upper-limb hemiparesis reported that combining NIBS with visual feedback yielded superior motor outcomes across multiple measures compared to monotherapies; however, no such investigations have focused on lower limb rehabilitation [11]. This systematic review aims to address this gap by evaluating current evidence on the scope of effect of combined NIBS and visual feedback in patients with lower limb hemiparesis, thereby assessing its potential role in future clinical practice and rehabilitation research.

## 2. Methods

### 2.1. Literature Review Design

This systematic review was registered with PROSPERO (CRD42024610688) and conducted following PRISMA guidelines.

### 2.2. Search Strategy

A comprehensive search of PubMed, Embase, Scopus, and Cochrane was conducted from 1 January 2000, to 1 November 2024, using terms associated with NIBS, VF, stroke, and motor rehabilitation. The following terms were searched for in titles, abstracts, and keywords:

(“brain computer interface” OR “visual feedback” OR “virtual reality” OR “virtual” OR “feedback” OR “vr” OR “brain-computer interface” OR “bci”) AND (“transcranial magnetic stimulation” OR “tms” OR “rtms” OR “noninvasive brain stimulation” OR “neuromodulation” OR “ transcranial direct current stimulation” OR “tDCS” OR “noninvasive brain stimulation” OR “noninvasive stimulation”) AND (“stroke” OR “cerebrovascular accident” OR “brain injury”) AND (“lower limb” OR “balance” OR “gait” OR “posture” OR “walking” OR “locomotion” OR “training” OR “lower limbs” OR “legs” OR “leg” OR “motor dysfunction” OR “motor impairment”).

### 2.3. Eligibility Criteria

Two authors (SN and LDR) independently reviewed the screened titles and abstracts, with any conflicts resolved by a third investigator (LDC). Authors of studies available only as abstracts were contacted for full texts. Reference lists of the included studies were manually reviewed to identify additional studies meeting the inclusion criteria.

Inclusion criteria were: (i) Studies involving adults (≥18 years) diagnosed with stroke of any stage (acute, subacute, chronic), presenting lower limb motor impairments; (ii) Studies using interventions that combine VF modalities (VR, BCI, mirror therapy) with tDCS or rTMS; (iii) Studies reporting lower limb functional outcomes, including but not limited to balance, gait, strength, or coordination, using validated measures; (vi) Studies with ≥5 patients; and (iv) Randomized controlled trials, prospective, or retrospective studies written in English.

The exclusion criteria were: (i) Studies involving pediatric populations or without age stratification; (ii) Studies not reporting lower limb motor outcomes or including non-stroke patients; (iii) Studies not combining VF and NIBS as interventions; (iv) Non-peer-reviewed literature, including reviews, editorials, abstracts; (v) Studies with <5 participants; and (vi) non-English publications.

### 2.4. Data Extraction

All data were independently extracted by two authors (SNC and FP), and a third independent author (LDC) addressed any discrepancies in the extracted data. Extracted variables included study design, participant demographics, intervention characteristics (including type, parameters, and timing of NIBS and VF), and reported outcomes related to motor function, balance, and gait. Data were then collated and organized into summary tables.

### 2.5. Quality Assessment

Two authors (JEC and FRC) independently assessed study quality after full-text screening. Randomized controlled trials (RCTs) were evaluated using the PEDro scale, and only studies scoring >6 were included. RCTs were subsequently assessed using the Cochrane RoB 2 tool for randomized trials and the Newcastle–Ottawa Scale for cohort studies.

## 3. Results

### 3.1. Study Selection

**Figure 1:**
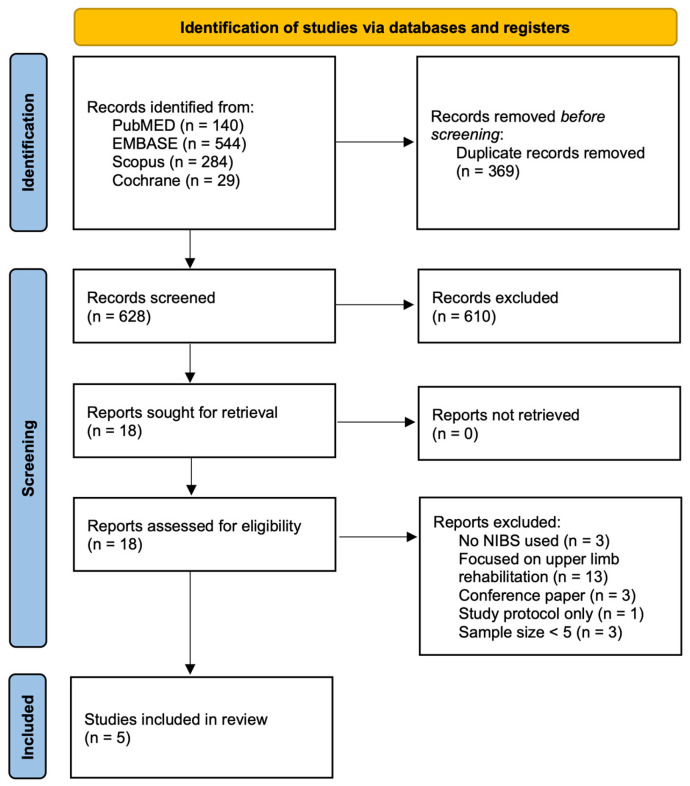
PRISMA flow chart describing article screening, selection, and extraction processes.

Our initial search identified 997 records across the four databases. After removing 373 duplicates, we screened the abstracts of the remaining 624 articles. A further 606 records were removed during the title and abstract screening. A total of 18 full-text articles were assessed for eligibility, and 13 were excluded for the following reasons: no NIBS reported (*n* = 3), focus on upper limb rehabilitation (*n* = 3), small sample size (*n* = 3), only the study protocol was availability (*n* = 1), and being conference abstracts (*n* = 3). Following full-text screening, 5 studies met the inclusion criteria, comprising 3 RCTs and 2 cohort studies (Figure 1).

### 3.2. Included Studies

#### Patient Demographics

The included studies exhibited substantial heterogeneity in participant characteristics, with sample sizes ranging from 5 to 66. Carlos et al. exclusively recruited ischemic stroke patients, while the remaining studies included both ischemic and hemorrhagic cases [16]. The interval between stroke onset and treatment initiation varied considerably, with most studies focusing on chronic stroke populations. An exception was Cha et al., who recruited patients within six months of stroke [17]. Patient characteristics were more rigorously controlled in studies by Cheng et al. and Qurat-ul-ain et al., both of which stratified participants based on stroke laterality [18,19]. These interstudy differences in patient characteristics are summarized in Table 1.

### 3.3. Intervention Protocols

#### 3.3.1. NIBS Parameters

The NIBS protocols varied significantly in terms of stimulation sites and parameters, including frequency, intensity, and duration. The moment of application differed between studies, with some delivering NIBS in parallel with functional training sessions, while others applied it in sequence (Table 2).

#### 3.3.2. Visual Feedback

VF protocols in the included studies utilized both immersive and non-immersive systems. Immersive VR systems using head-mounted displays incorporate obstacle navigation and spatial awareness tasks as exercises for motor learning [16,20]. Non-immersive approaches included added mirror therapy and non-immersive virtual feedback, employing motion detection and projecting it on a stationary display (Table 3) [17,18,19].

### 3.4. Risk of Bias

Multiple tools were used to assess the bias in the included studies. The three RCTs included in this review were assessed using the Cochrane Risk of Bias 2.0 tool and the PEDro scale. All studies employed reported clear randomization protocols and predefined outcomes. However, incomplete reporting of blinding—particularly of therapists—introduced potential sources of bias. Additionally, follow-ups were relatively short (Figure S1 and Table S2).

The two cohort studies were evaluated using the NOS, each receiving a score of 6 out of 9. Both studies demonstrated strong participant selection and reported outcomes with adequate follow-up. However, both studies reported small sample sizes, which reduced the comparability and generalizability of their findings (Table S3).

### 3.5. Outcomes

The included studies employed a range of tools to assess gait improvement, balance, and motor performance. Additionally, some studies incorporated neurophysiological parameters, such as EEG features and corticospinal excitability, to provide further insight into the effects of the intervention (Table S1).

Baseline motor abilities varied considerably across and within studies, reflecting the heterogeneity of participant populations. Carlos et al. reported moderate baseline motor abilities (TUG: 14.4 s) [16], while Salameh et al.’s cohort showed more severe impairment (TUG: 36.8 s) (Table 4) [20]. In Cheng et al., baseline FMA scores in the combined visual feedback and sham group (19.7 ± 8.7) were significantly lower than those in the visual feedback and rTMS (25.1 ± 9.2) and the conventional therapy and sham groups (24.9 ± 7.1) [18]. Similarly, Qurat-ul-ain et al. noted that their sham group exhibited better motor function at baseline than both intervention arms [19]. These imbalances complicate cross-study comparisons and raise concerns about the internal validity of their analyses.

#### 3.5.1. Balance

Balance was reported across 3 of the included studies and was primarily assessed using the BBS score. Carlos et al. reported a significant increase in BBS from 43.6 to 47.5 following XR training and tDCS (Table 4) [16]. Among the RCTs, Cheng et al. reported the only significant improvement in BBS scores when visual feedback was combined with rTMS (*p* = 0.011). In contrast, both sham stimulation (*p* = 0.066) and sham combined with conventional therapy (*p* = 0.109) resulted in inferior outcomes [18]. Similarly, Qurat-ul-ain et al. reported the most significant BBS improvements when combining motor cortex tDCS stimulation with VF group, followed by the same protocol with cerebellar stimulation [19]. The group receiving VF and sham stimulation experienced no significant changes. Not only does this demonstrate the potential additive effect of combining NIBS with VF, but it also highlights the influence of the stimulation site as a modifiable factor for therapeutic efficacy.

Furthermore, additional measures for balance were reported across the included studies. Cha et al. evaluated postural sway as an indicator of balance control. Both experimental groups demonstrated significant reductions in sway following intervention with mirror therapy (*p* < 0.05), regardless of whether rTMS stimulation was present. However, the rTMS stimulation group exhibited a larger degree of effect than the sham control (−32.0 mm vs. −21.7 mm, *p* < 0.05) [17]. Additionally, Qurat-ul-ain et al. reported significant improvements in reactive postural responses (BESTest-RPR) following both cerebellar and M1 tDCS compared to sham but reporting no difference between the effective stimulation sites [19].

#### 3.5.2. Gait Performance

Gait performance was reported in all included studies, with the most common measures of gait performance employed being the TUG test and 6 MWT. Cheng et al. reported the most significant improvement in TUG in the group undergoing VF combined with rTMS (−9.4 ± 17.8 s; *p* = 0.008), outperforming both the groups with VF and conventional therapy with sham (−2.2 ± 4.3 s, *p* = 0.093; −0.2 ± 1.6 s, *p* = 0.541) (Table 4) [18]. Similarly, Qurat-ul-ain et al. observed the most substantial TUG gains in the cerebellar tDCS group (–5.03 s), followed by motor cortex stimulation (−3.27 s) and sham (−1.7 s) [19]. In the same cohorts, there was no significant improvement in the 6 MWT distances. Cha et al. found similar directions of effect in both TUG and 6 MWT using mirror therapy with and without rTMS (*p* < 0.05). However, the size of the effect was larger in the combined rTMS intervention [17]. Using an additional metric, similar results were observed on the Wisconsin Gait Scale. Across all outcomes, intergroup *p*-values were not reported, limiting the ability to quantify the statistical significance of between-group differences.

Additionally, Salameh et al. reported significant improvements in gait speed following ten sessions of combined VR and tDCS, with mean changes from baseline of +0.25 m/s post-treatment (95% CI: 0.11, 0.40; *p* = 0.01) and +0.27 m/s at final follow-up (95% CI: 0.12, 0.42; *p* = 0.01) [20].

#### 3.5.3. Motor Performance

The FMA is a stroke-specific, performance-based impairment index measuring motor performance, sensation, balance, range of motion, and pain. It was reported in 3 of the included studies. The results from the included studies were mixed, with Carlos et al. and Salameh et al. reporting significant improvements following tDCS and VR training (*p* = 0.001) (Table 4) [16,20]. In contrast, Cheng et al. found no significant differences in FMA scores in the combined visual feedback and rTMS (*p* = 0.102), visual feedback and sham (*p* = 0.102), and conventional therapy and sham groups (*p* = 0.317) [18].

#### 3.5.4. Further Outcomes

Cheng et al. reported that the combined intervention of VF and rTMS led to increased cortical excitability, modulation across both hemispheres, and improved symmetry in motor evoked potential (MEP) latency. Improvements were observed even in patients with no measurable MEPs at baseline, including one who showed newly detectable responses post-intervention. These findings suggest that rTMS applied over the unaffected hemisphere may enhance bilateral corticospinal excitability, reinforcing the neuroplastic benefits of combined therapy [18]. Similarly, Carlos et al. conducted a symmetry analysis using EEG-based Brain Symmetry Index (BSI) metrics, observing progressive reductions in hemispheric imbalance across rehabilitation sessions. These shifts toward greater symmetry, particularly in beta and gamma bands, were interpreted as neurophysiological markers of neuroplasticity and paralleled improvements in lower limb function within the combined XR and tDCS group [16].

## 4. Discussion

Lower limb motor impairment represents one of the most persistent and debilitating outcomes of stroke. Recent technological advances have presented potential treatment options for this frequently refractory condition. While VF and NIBS have demonstrated their efficacy as individual therapies, their combined impact in rehabilitation is currently under investigation [21,22]. This systematic review is the first to examine the application of VF and NIBS in the context of lower limb rehabilitation in stroke patients.

Overall, the literature regarding the efficacy of this combination therapy in lower limb rehabilitation is conflicting. Among outcome domains, balance showed the most consistent improvement, particularly when VF was paired with either tDCS or rTMS, suggesting a potential additive effect of combined interventions on postural control. Outcomes regarding gait were more variable: while TUG times improved in several trials, the effects of the intervention on 6 MWT were inconsistent, and statistical analyses referencing intergroup comparisons were often absent. Motor performance results were mixed, with no RCTs reporting superiority in FMA improvements following combination therapy as compared to conventional or virtual reality therapies with sham [18]. To explore how NIBS mechanisms may relate to clinical outcomes, several studies have explored electrophysiological transformations within this cohort, demonstrating increased cortical excitability and hemispheric symmetry, which are presented as biomarkers of underlying neuroplastic effects. However, the extent to which these changes confer clinical improvements remains unclear.

The literature focusing on the combined use of VF and NIBS therapy for upper limb motor function after stroke is more established [23]. A recent meta-analysis including 493 patients across 11 studies found significant improvements in FMA, muscle spasticity, and daily living ability when combining NIBS and VR treatment compared to traditional or single training methods [11]. In their analysis, the authors aimed to identify which parameters in the interventions were linked to superior outcomes; however, substantial heterogeneity in the protocols across studies prevented them from drawing conclusions. To address this gap, the following section investigates how specific intervention characteristics have been suggested to influence treatment efficacy in the literature.

The included studies utilized both immersive and non-immersive feedback systems; however, the relationship between the degree of immersion and clinical improvement could not be determined. From these studies, only Carlos et al. and Salameh et al. utilized fully immersive systems, reporting significant improvements in balance, gait, and motor function [16,20]. However, the absence of a comparator limits conclusions about their relative efficacy compared to conventional therapy and in combination with NIBS. All remaining RCTs applied non-immersive VR or mirror therapy protocols.

This is consistent with prior systematic reviews suggesting that VF systems, regardless of immersion depth, can induce neuroplastic changes and improve cortical connectivity [24]. Immersive VR may mediate motor recovery by stimulating experience-dependent plasticity through neurocognitive strategies, such as motor imagery and action observation, which activate mirror neurons to develop motor representations of actions at both neural and behavioral levels [25,26,27]. A recent network meta-analysis of 20 RCTs found immersive VR to be more effective in enhancing upper limb function than non-immersive platforms and conventional therapy [28]. However, no comparable synthesis has been conducted for lower limb outcomes, reflecting the limited research in this area.

Additionally, immersive VR has been associated with improved patient adherence to therapy, which is particularly relevant in lower limb rehabilitation, where the population is typically affected by poor mental health and motivation, which impede sustained engagement [29,30].

Furthermore, a challenge for the more widespread clinical implementation of NIBS has been the variability in parameters, including stimulation polarity, intensity, duration, electrode placement, and the timing of stimulation in relation to behavioral training tasks [31]. Across the five included trials, three used tDCS and two used rTMS, with stimulation targeting ipsilesional M1, contralesional M1, or the cerebellum. tDCS studies varied further in electrode polarity. From these, the most effective location for stimulation could not be elucidated. However, a recent meta-analysis reported that cathodal or anodal tDCS stimulation of the unaffected M1 cortex yielded significant interventional effects in upper limb rehabilitation in combination with VR [11]. In contrast, stimulation of the affected hemisphere did not [11]. These findings suggest that stimulation site and polarity may play a role in the efficacy of NIBS in this patient population, with emerging evidence suggesting that targeting the unaffected hemisphere may enhance compensatory activity in the affected hemisphere [32]. Future research should prioritize identifying the most effective dose–response relationship to optimize therapeutic outcomes.

Intervention protocols varied considerably across studies, with durations ranging from 3 days to 4 weeks, most commonly spanning 2 to 4 weeks with five sessions per week. The timing of NIBS relative to VR therapy also differed, with most studies applying stimulation concurrently. Session durations for tDCS and rTMS ranged from 15–30 min and 10–15 min, respectively. In contrast, stimulation intensities were consistent, with all tDCS interventions using 2 mA and all rTMS interventions employing 1 Hz protocols. However, no study systematically evaluated how these protocol variations influence treatment efficacy, highlighting an important area for further investigation.

Beyond protocol efficacy, cost-effectiveness and logistical feasibility represent major barriers to the broader implementation of combined NIBS and VF interventions. High equipment costs, necessary operator training, and the need for specialized infrastructure—particularly for immersive VR systems—limit their integration into standard clinical practice. To date, economic evaluations and implementation studies are scarce, although they will be central when determining the feasibility and scalability of this therapeutic approach.

Taken together, while preliminary evidence points to promising effects, these unaddressed gaps highlight the need for further investigations to be conducted before routine clinical use can be recommended.

## 5. Limitations

Our study has several limitations. Firstly, our analysis included patients at various stages of stroke. This has been shown to be a mediating factor in the effectiveness of motor rehabilitation following stroke [33]. Excluding one study, all papers included patients 6 months after their stroke event, thereby representing a primarily chronic stroke cohort, limiting the generalizability of our results to patients in the acute and subacute phases of recovery. Secondly, the included studies did not stratify results based on stroke etiology (ischemic vs. hemorrhagic), despite growing interest in their differential response to therapy [34,35]. As a result, conclusions regarding the efficacy of combined NIBS and VF in these subpopulations remain unclear. Third, all included papers excluded patients with significant cognitive impairments, thereby overlooking an important subset of stroke survivors and further limiting generalizability.

Furthermore, follow-ups across the included studies were relatively brief, with outcomes reported immediately after or proximal to their final sessions, providing limited insight into the permanence of recovered motor gains. Another overlooked factor was the reporting of whether participants had experienced their first stroke or if they experienced recurrent strokes, as this could significantly influence their baseline recovery potential and responsiveness to treatment [36,37]. Additionally, the included studies lacked reporting on stroke severity and lesion location beyond hemispheric involvement, both of which are important determinants of motor recovery potential and may act as confounding factors.

Additionally, the lack of standardized protocols and inconsistent comparator groups across studies reduces the reliability of efficacy comparisons. Interventions were assessed against various benchmarks, including conventional therapy, sham stimulation, or no treatment, resulting in inconsistent reports of benefits. These methodological challenges, coupled with the typically small sample sizes of the included studies, emphasize the need for more rigorous and standardized research designs in this field of study.

Finally, due to the small number of included studies, we were unable to formally assess publication bias. Moreover, the potential for selective outcome reporting could not be evaluated, as most studies lacked registered protocols and frequently reported multiple outcomes.

## 6. Conclusions

The integration of NIBS and VF has shown preliminary promise across various domains of stroke rehabilitation. This systematic review is the first to evaluate the current evidence supporting its efficacy in the context of lower limb motor recovery in stroke patients. Preliminary evidence suggests that combining these interventions may be associated with improvements in motor function, balance, and gait performance compared to conventional rehabilitation or either intervention alone.

While these findings are promising, they must be interpreted with caution due to the substantial heterogeneity across the included studies in terms of intervention, timing, duration, stimulation parameters, and outcome measures. Additionally, the current body of evidence is limited by the small number of studies and small sample sizes, leaving several gaps that require further investigation.

To better understand the effects of this combination therapy, future research should build upon this preliminary evidence, designing more targeted treatment protocols, assessing the long-term durability of outcomes, and investigating the mechanisms underlying functional recovery across differing populations of stroke patients, evaluating its potential role both as a first-line rehabilitation strategy as well as an adjunct for patients refractory to conventional therapy. Addressing these gaps will be central to determine whether this approach can be translated into effective clinical interventions that ultimately improve the quality of life in stroke patients.

## Figures and Tables

**Table 1:** This table provides a comprehensive overview of the included studies, outlining variations in demographic composition, stroke characteristics, and treatment timelines.

Author and Year	Country	Sample Size (Intervention/ Control)	Mean Age (±SD)	Sex Ratio (M/F)	Stroke Characteristics (Ischemic/Hemorrhagic)	Interval Stroke-Treatment (Months)	Side of Stroke (R/L)
Carlos et al. (2024) [16]	Brazil	10/NA	60 ± 12	6/4	10/0	32 ± 23	7/3
Cha et al. (2017) [17]	Republic of Korea	15/15	62.67 ± 6.99	15/15	19/11	<6	NA
Cheng et al. (2023) [18]	Taiwan	(10 + 10)/10	59.93 ± 15.43	19/11	NA	36.47 ± 24.71	15/15
Qurat-ul-ain et al. (2022) [19]	Pakistan	(22 + 22)/22	57.48 ± 5.99	54/12	50/16	NA	33/33
Salameh et al. (2022) [20]	USA	5/NA	58.6 ± 7	5/0	3/5	68	1/4

Abbreviations: NA: not available.

**Table 2:** This table illustrates the NIBS protocols across studies, including differences in stimulation sites, frequencies, and the temporal relationship to other rehabilitation interventions.

Author	Type of NIBS Utilized	Stimulation Site	Intensity and Duration	Frequency	Temporal Relation
Carlos et al. (2024) [16]	Anodal tDCS	Ipsilesional M1	2 mA current, with a ramp-up and ramp-down time of 30 s, for 30 min per session.	10 sessions over 2 weeks, every morning on weekdays.	In parallel; tDCS administered throughout the entire session.
Cha et al. (2017) [17]	rTMS	Experimental group: Cerebellum Sham group: rTMS on irrelevant brain localization	1 Hz, for 15 min	20 sessions, once a day, 5 times a week for a period of 4 weeks.	In series: rTMS was performed before mirror therapy.
Cheng et al. (2023) [18]	rTMS	Experimental group: Contralesional M1 Sham group: perpendicularly to the scalp and the same stimulus intensity and pattern.	1 Hz, for 10 min	3 sessions per week for 4 weeks.	In series: rTMS administered for 10 min before 40 min VF training
Qurat-ul-ain et al. (2022) [19]	Anodal + Cathodal tDCS	Cerebellar stimulation group (CbSG): Anode, ipsilesional cerebellum + Cathode, contralesional cerebellum M1 Stimulation Group (MSG): Anode, ipsilesional M1 + Cathode, contralesional M1 No sham group	2 mA current, stable over 20 min	3 consecutive training sessions over 3 days.	In parallel: tDCS administered throughout the session.
Salameh et al. (2022) [20]	Anodal + Cathodal tDCS	Anode: ipsilesional M1 Cathode: homologous contralesional M1	2 mA current, stable over 15 min	10 gait training sessions over 2 weeks	In parallel: tDCS administered over the first 15 min of the phase one treadmill VR training. In phase two, no tDCS was used (30 min of overground training)

**Table 3:** This table outlines the visual feedback modalities and protocols across studies.

Author, Year	Type of Visual Feedback	Qualitative Description of Feedback Protocol
Carlos et al. (2024) [16]	XR	Immersive VR system, “GestureCollection”, including stationary marches with obstacle tasks to improve spatial awareness, followed by a five-minute obstacle-free march in a Google Maps-based environment.
Cha et al. (2017) [17]	Mirror Therapy	Participants visualized their reflected image while performing tasks.
Cheng et al. (2023) [18]	Game-Based VR	Developed an ankle haptic exercise program integrated with a flying video game where the patient’s paretic ankle controlled an aircraft presented on a screen.
Qurat-ul-ain et al. (2022) [19]	Xbox Kinect	Xbox Kinect sessions for lower limb rehabilitation through games, like soccer and basketball. Infrared tracking tracked active movements.
Salameh et al. (2022) [20]	VR	V-Gait VR system, a treadmill-based setup where users navigate obstacles while receiving audio-visual feedback.

**Table 4:** This table summarizes outcome measures across studies, evaluating the differences between pre-therapeutic and post-therapeutic scores for balance, gait, and motor dysfunction. Results in bold indicate significant results.

Author and Year	Group	Balance (BBS)		Gait (TUG/6 MWT)		Motor Function (FMA)		Assessment Timing
		Pre-Therapy	Post-Therapy	Pre-Therapy	Post-Therapy	Pre-Therapy	Post-Therapy	
Carlos et al. (2024) [16]	XR training + tDCS	43.6	47.5	TUG: 36.8	TUG: 32.0	24.2	26.3	T0: Before first session T1: After final session
Cha et al. (2017) [17]	Mirror therapy + rTMS	NR	NR	TUG: 30.40 ± 4.29 6 MWT: 120.67 ± 25.67	TUG: 24.47 ± 4.55 6 MWT: 181.47 ± 34.52	NR	NR	T0: Before first session T1: After 4 weeks of training.
	Mirror therapy + Sham	NR	NR	TUG: 31.60 ± 3.56 6 MWT: 118.24 ± 30.84	TUG: 28.93 ± 3.13 6 MWT: 165.72 ± 35.63	NR	NR	
Cheng et al. (2023) [18]	Non-immersive VR+ rTMS	41.7 ± 11.5	43.6 ± 10.9	TUG: 39.3 ± 32.2	TUG: 29.8 ± 17.2	25.1 ± 9.2	25.6 ± 8.9	T0: 1 day before first session T1: 1 day after final session
	Non-immersive VR + Sham	34.7 ± 13.8	37.5 ± 14.9	TUG: 51.4 ± 40.2	TUG: 49.1 ± 40.2	19.7 ± 8.7	20.6 ± 8.9	
	Conventional training + Sham	40.3 ± 18.2	41.4 ± 18.5	TUG: 30.8 ± 23.9	TUG: 30.5 ± 39.4	24.9 ± 7.1	25.0 ± 7.0	
Qurat-ul-ain et al. (2022) [19]	Non-immersive VR + Cerebellar tDCS	41.63 ± 8.2	47.91 ± 7.7	TUG: 17.54 ± 7.6 6 MWT: 0.12 ± 0.03	TUG: 12.51 ± 6.3 6 MWT: 0.18 ± 0.04	NR	NR	T0: Before first session T1: After 3 sessions
	Non-immersive VR + M1 tDCS	40.27 ± 8.4	49.36 ± 3.6	TUG: 13.85 ± 4.9 6 MWT: 0.13 ± 0.03	TUG: 10.58 ± 4.6 6 MWT: 0.17 ± 0.05	NR	NR	
	Non-immersive VR + Sham	50.27 ± 3.1	52.36 ± 3.0	TUG: 9.69 ± 2.6 6 MWT: 0.17 ± 0.8	TUG: 7.99 ± 2.4 6 MWT: 0.23 ± 0.09	NR	NR	
Salameh et al. (2022) [20]	VR training + tDCS	NR	NR	TUG: 14.40 ± 7.49	TUG: 12.06 ± 6.74	21.2 ± 4.5	25.40 ± 3.21	T0: Before first session T1: 3-week follow up

Abbreviations: BBS: Berg Balance Scale, FMA: Fugl–Meyer Assessment, 6 MWT: 6-Minute Walk Test, rTMS: Repetitive Transcranial Magnetic Stimulation, tDCS: Transcranial Direct Current Stimulation, XR: Extended Reality, TUG: Timed Up and Go, VR: Virtual Reality.

## Data Availability

No new data were created or analyzed in this study. Data sharing is not applicable to this article.

## Supplementary Materials

The following supporting information can be downloaded at: https://www.mdpi.com/article/10.3390/jcm14145027/s1, Table S1: This table outlines the primary outcomes measured across studies, Figure S1: Summary plot of RoB2 assessments for all included RCTs, Table S2: Table summarizing the PEDro scale scores for all included RCTs, Table S3. Table summarizing the NOS scores for included cohort studies.

## References

[1] Tsao C.W., Aday A.W., Almarzooq Z.I., Alonso A., Beaton A.Z., Bittencourt M.S., Boehme A.K., Buxton A.E., Carson A.P., Commodore-Mensah Y. (2022). Heart Disease and Stroke Statistics—2022 Update: A Report from the American Heart Association. Circulation.

[2] Feigin V.L., Roth G.A., Naghavi M., Parmar P., Krishnamurthi R., Chugh S., Mensah G.A., Norrving B., Shiue I., Ng M. (2016). Global burden of stroke and risk factors in 188 countries, during 1990–2013: A systematic analysis for the Global Burden of Disease Study 2013. Lancet Neurol..

[3] Krishnamurthi R.V., Feigin V.L., Forouzanfar M.H., Mensah G.A., Connor M., Bennett D.A., Moran A.E., Sacco R.L., Anderson L.M., Truelsen T. (2013). Global and regional burden of first-ever ischaemic and haemorrhagic stroke during 1990–2010: Findings from the Global Burden of Disease Study 2010. Lancet Glob. Health.

[4] Roger V.L., Go A.S., Lloyd-Jones D.M., Adams R.J., Berry J.D., Brown T.M., Carnethon M.R., Dai S., De Simone G., Ford E.S. (2011). Heart Disease and Stroke Statistics—2011 Update: A Report from the American Heart Association. Circulation.

[5] Cauraugh J. (2003). Chronic stroke motor recovery: Duration of active neuromuscular stimulation. J. Neurol. Sci..

[6] Sathian K., Buxbaum L.J., Cohen L.G., Krakauer J.W., Lang C.E., Corbetta M., Fitzpatrick S.M. (2011). Neurological Principles and Rehabilitation of Action Disorders: Common Clinical Deficits. Neurorehabilit. Neural Repair.

[7] Kleim J.A., Jones T.A. (2008). Principles of experience-dependent neural plasticity: Implications for rehabilitation after brain damage. J. Speech Lang. Hear. Res. JSLHR.

[8] Mekbib D.B., Han J., Zhang L., Fang S., Jiang H., Zhu J., Roe A.W., Xu D. (2020). Virtual reality therapy for upper limb rehabilitation in patients with stroke: A meta-analysis of randomized clinical trials. Brain Inj..

[9] Soleimani M., Ghazisaeedi M., Heydari S. (2024). The efficacy of virtual reality for upper limb rehabilitation in stroke patients: A systematic review and meta-analysis. BMC Med. Inform. Decis. Mak..

[10] Potcovaru C.-G., Cinteză D., Săndulescu M.I., Poenaru D., Chiriac O., Lambru C., Moldoveanu A., Anghel A.M., Berteanu M. (2024). The Impact of Virtual Reality as a Rehabilitation Method Using TRAVEE System on Functional Outcomes and Disability in Stroke Patients: A Pilot Study. Biomedicines.

[11] Zhang N., Wang H., Wang H., Qie S. (2024). Impact of the combination of virtual reality and noninvasive brain stimulation on the upper limb motor function of stroke patients: A systematic review and meta-analysis. J. NeuroEng. Rehabil..

[12] Orrù G., Conversano C., Hitchcott P.K., Gemignani A. (2020). Motor stroke recovery after tDCS: A systematic review. Rev. Neurosci..

[13] González-Rodriguez B., Serradell-Ribé N., Viejo-Sobera R., Romero-Muñoz J.P., Marron E.M. (2022). Transcranial direct current stimulation in neglect rehabilitation after stroke: A systematic review. J. Neurol..

[14] Hara T., Shanmugalingam A., McIntyre A., Burhan A.M. (2021). The Effect of Non-Invasive Brain Stimulation (NIBS) on Attention and Memory Function in Stroke Rehabilitation Patients: A Systematic Review and Meta-Analysis. Diagnostics.

[15] Tang X., Zhang N., Shen Z., Guo X., Xing J., Tian S., Xing Y. (2024). Transcranial direct current stimulation for upper extremity motor dysfunction in poststroke patients: A systematic review and meta-analysis. Clin. Rehabil..

[16] Carlos B.M., Menezes L.T., Rosa B., Furumoto B.F., Feitosa S.S., Fernandes C.A., Ferreira-Melo S.E., Pereira J.D., Almeida S., Brandão A.F. (2024). Graph network and symmetry analysis after combined XR and tDCS in stroke rehabilitation. Biomed. Signal Process. Control.

[17] Cha H.G. (2017). The Effect of Low-Frequency (1 Hz) rTMS on the Cerebellar Cortex in Patients with Ataxia After a Posterior Circulation Stroke: Randomized Control Trial. J. Magn..

[18] Cheng H.L., Lin C.H., Tseng S.H., Peng C.W., Lai C.H. (2023). Effectiveness of Repetitive Transcranial Magnetic Stimulation Combined with Visual Feedback Training in Improving Neuroplasticity and Lower Limb Function after Chronic Stroke: A Pilot Study. Biology.

[19] Qurat-ul-ain, Ahmad Z., Ishtiaq S., Ilyas S., Shahid I., Tariq I., Malik A.N., Liu T., Wang J. (2022). Short term effects of anodal cerebellar vs. anodal cerebral transcranial direct current stimulation in stroke patients, a randomized control trial. Front. Neurosci..

[20] Salameh A., McCabe J., Skelly M., Duncan K.R., Chen Z., Tatsuoka C., Bikson M., Hardin E.C., Daly J.J., Pundik S. (2022). Stance Phase Gait Training Post Stroke Using Simultaneous Transcranial Direct Current Stimulation and Motor Learning-Based Virtual Reality-Assisted Therapy: Protocol Development and Initial Testing. Brain Sci..

[21] Invernizzi M., Negrini S., Carda S., Lanzotti L., Cisari C., Baricich A. The Value of Adding Mirror Therapy for Upper Limb Motor Recovery of Subacute Stroke Patients: A Randomized Controlled Trial. Published Online 2013. https://air.unimi.it/handle/2434/721399.

[22] Chen J.M., Li X.L., Pan Q.H., Yang Y., Xu S.M., Xu J.W. (2023). Effects of non-invasive brain stimulation on motor function after spinal cord injury: A systematic review and meta-analysis. J. NeuroEng. Rehabil..

[23] Donati D., Pinotti E., Mantovani M., Casarotti S., Fini A., Tedeschi R., Caselli S. (2025). The Role of Immersive Virtual Reality in Upper Limb Rehabilitation for Subacute Stroke: A Review. J. Clin. Med..

[24] Hao J., Xie H., Harp K., Chen Z., Siu K.C. (2022). Effects of Virtual Reality Intervention on Neural Plasticity in Stroke Rehabilitation: A Systematic Review. Arch. Phys. Med. Rehabil..

[25] Thieme H., Morkisch N., Mehrholz J., Pohl M., Behrens J., Borgetto B., Dohle C. Mirror Therapy for Improving Motor Function After Stroke—Thieme, H—2018|Cochrane Library. https://www.cochranelibrary.com/cdsr/doi/10.1002/14651858.CD008449.pub3/full.

[26] Ramachandran V.S., Altschuler E.L. (2009). The use of visual feedback, in particular mirror visual feedback, in restoring brain function. Brain J. Neurol..

[27] Jeannerod M. (2001). Neural Simulation of Action: A Unifying Mechanism for Motor Cognition. NeuroImage.

[28] Hao J., He Z., Yu X., Remis A. (2023). Comparison of immersive and non-immersive virtual reality for upper extremity functional recovery in patients with stroke: A systematic review and network meta-analysis. Neurol. Sci..

[29] Terrill A.L., Schwartz J.K., Belagaje S.R. (2018). Best Practices for The Interdisciplinary Rehabilitation Team: A Review of Mental Health Issues in Mild Stroke Survivors. Stroke Res. Treat..

[30] De Rooij I.J.M., van de Port I.G.L., Punt M., Moorsel P.J.M.A.-V., Kortsmit M., van Eijk R.P.A., Visser-Meily J.M.A., Meijer J.-W.G. (2021). Effect of Virtual Reality Gait Training on Participation in Survivors of Subacute Stroke: A Randomized Controlled Trial. Phys. Ther..

[31] Antal A., Luber B., Brem A.-K., Bikson M., Brunoni A.R., Kadosh R.C., Dubljević V., Fecteau S., Ferreri F., Flöel A. (2022). Non-invasive brain stimulation and neuroenhancement. Clin. Neurophysiol. Pract..

[32] Wang Q., Zhang D., Zhao Y.Y., Hai H., Ma Y.W. (2020). Effects of high-frequency repetitive transcranial magnetic stimulation over the contralesional motor cortex on motor recovery in severe hemiplegic stroke: A randomized clinical trial. Brain Stimul..

[33] Hu J., Du J., Xu Q., Yang F., Zeng F., Weng Y., Dai X.-J., Qi R., Liu X., Lu G. (2018). Dynamic Network Analysis Reveals Altered Temporal Variability in Brain Regions after Stroke: A Longitudinal Resting-State fMRI Study. Neural Plast..

[34] Oosterveer D.M., Wermer M.J.H., Volker G., Vlieland T.P.M.V. (2022). Are There Differences in Long-Term Functioning and Recovery Between Hemorrhagic and Ischemic Stroke Patients Receiving Rehabilitation?. J. Stroke Cerebrovasc. Dis..

[35] Perna R., Temple J. (2015). Rehabilitation Outcomes: Ischemic versus Hemorrhagic Strokes. Behav. Neurol..

[36] San X., Lv Z., Xu P., Wang J., Lan T. (2022). The prevention of stroke by statins: A meta-analysis. Medicine.

[37] Chaudhary D., Khan A., Shahjouei S., Gupta M., Lambert C., Avula V., Schirmer C.M., Holland N., Griessenauer C.J., Azarpazhooh M.R. (2021). Trends in ischemic stroke outcomes in a rural population in the United States. J. Neurol. Sci..

