# Supplementary material for: Combining Visual Feedback and Noninvasive Brain Stimulation for Lower Limb Motor Rehabilitation in Stroke: A Systematic Review of the Current Evidence
Source: J Clin Med. 2025 Jul 16;14(14):5027. doi: 10.3390/jcm14145027 (PMC12294890; doi:10.3390/jcm14145027)
Supplement: Supplementary file 1 [file jcm-14-05027-s001.zip › jcm-3763267-supplementary.pdf]

Supplementary Materials

Table S1. This table outlines the primary outcomes measured across studies.

Author and Year	Primary Measures	Outcome Measures
Carlos et al., 2024	Balance, motor performance, EEG characteristics' improvement	FMA<br>BBS<br>TUG
Salameh et al., 2022	Gait improvement	FMA<br>Gait Speed<br>TUG<br>FGA
Cha et al., 2017	Balance, gait improvement	Postural Sway<br>Wisconsin Gait Scale<br>TUG<br>6MWT
Cheng et al., 2023	Motor performance, gait improvement, balance, and corticospinal excitability	FMA<br>MEP<br>BBS<br>TUG
Qurat-ul-ain et al., 2022	Balance, gait and risk of fall improvement	BBS<br>BESTest<br>MMSE<br>MoCA<br>JHRAT<br>25FWT<br>6MWT

FMA: Fugl-Meyer Assessment
BBS: Berg Balance Scale
TUG: Timed Up and Go
FGA: Functional Gait Assessment
6MWT: 6-Minute Walk Test
MEP: Motor Evoked Potentials
BESTest: Balance Evaluation Systems Test
MMSE: Mini-Mental State Examination
MoCA: Montreal Cognitive Assessment
JHRAT: Johns Hopkins Risk Assessment Tool
25FWT: 25-Foot Walk Test

Figure S1. Summary plot of RoB2 assessments for all included RCTs.

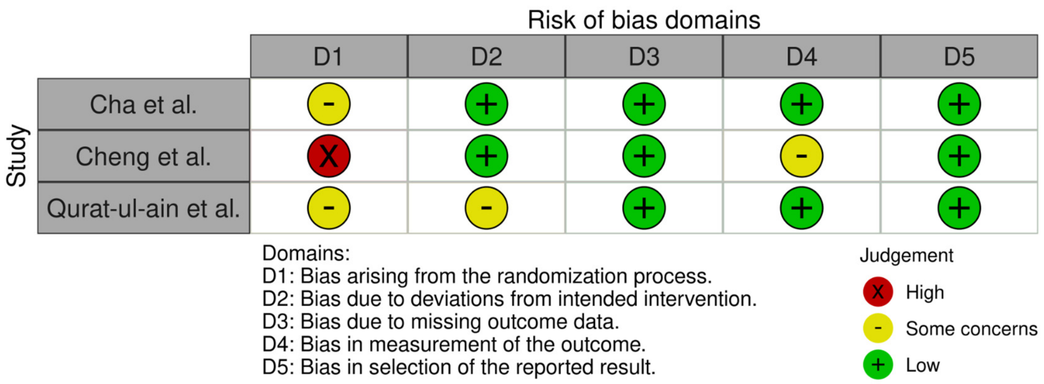

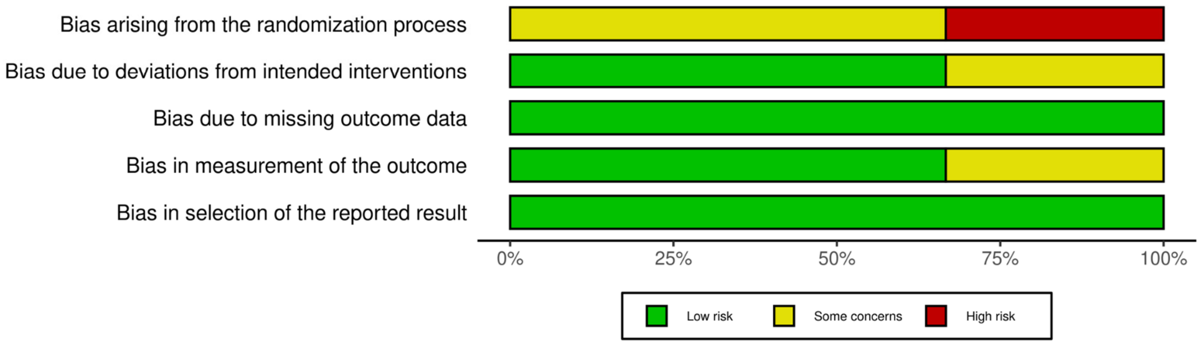

Table S2. Table summarizing the PEDro scale scores for all included RCTs.

Article, Year	Eligibility criteria	Random allocation	Allocation concealment	Baseline comparability	Blinding of subjects	Blinding of therapists	Blinding of assessors	Adequate follow-up	Intention to treat analysis	Between-group statistical comparison	Point measures and variability	Total Score
Cha et al., 2017	Y	Y	Y	Y	Y	N	Y	Y	Y	Y	Y	9/10
Cheng et al., 2023	Y	Y	N	Y	Y	N	Y	Y	Y	Y	Y	8/10
Qurat-ul-ain et al., 2022	Y	Y	N	Y	Y	N	Y	Y	Y	Y	Y	8/10

Table S3. Table summarizing the NOS scores for included cohort studies.

Article	Selection	Comparability	Outcome	Total
Carlos et al.	3	0	3	6/9
Salameh et al.	3	0	3	6/9
